# Micro- and Macro-Geographic Scale Effect on the Molecular Imprint of Selection and Adaptation in Norway Spruce

**DOI:** 10.1371/journal.pone.0115499

**Published:** 2014-12-31

**Authors:** Marta Scalfi, Elena Mosca, Erica Adele Di Pierro, Michela Troggio, Giovanni Giuseppe Vendramin, Christoph Sperisen, Nicola La Porta, David B. Neale

**Affiliations:** 1 Research and Innovation Centre, Fondazione Edmund Mach (FEM), S. Michele all'Adige, Trento, Italy; 2 National Research Council, Institute of Biosciences and Bioresources, Sesto Fiorentino, Firenze, Italy; 3 Swiss Federal Institute for Forest, Snow and Landscape Research (WSL), Birmensdorf, Switzerland; 4 Department of Plant Sciences, University of California Davis, Davis, CA, United States of America; Università Politecnica delle Marche, Italy

## Abstract

Forest tree species of temperate and boreal regions have undergone a long history of demographic changes and evolutionary adaptations. The main objective of this study was to detect signals of selection in Norway spruce (*Picea abies* [L.] Karst), at different sampling-scales and to investigate, accounting for population structure, the effect of environment on species genetic diversity. A total of 384 single nucleotide polymorphisms (SNPs) representing 290 genes were genotyped at two geographic scales: across 12 populations distributed along two altitudinal-transects in the Alps (micro-geographic scale), and across 27 populations belonging to the range of Norway spruce in central and south-east Europe (macro-geographic scale). At the macrogeographic scale, principal component analysis combined with Bayesian clustering revealed three major clusters, corresponding to the main areas of southern spruce occurrence, i.e. the Alps, Carpathians, and Hercynia. The populations along the altitudinal transects were not differentiated. To assess the role of selection in structuring genetic variation, we applied a Bayesian and coalescent-based *F*_ST_-outlier method and tested for correlations between allele frequencies and climatic variables using regression analyses. At the macro-geographic scale, the *F*_ST_-outlier methods detected together 11 *F*_ST_-outliers. Six outliers were detected when the same analyses were carried out taking into account the genetic structure. Regression analyses with population structure correction resulted in the identification of two (micro-geographic scale) and 38 SNPs (macro-geographic scale) significantly correlated with temperature and/or precipitation. Six of these loci overlapped with *F*_ST_-outliers, among them two loci encoding an enzyme involved in riboflavin biosynthesis and a sucrose synthase. The results of this study indicate a strong relationship between genetic and environmental variation at both geographic scales. It also suggests that an integrative approach combining different outlier detection methods and population sampling at different geographic scales is useful to identify loci potentially involved in adaptation.

## Introduction

Adaptation of forest tree species to their environment is of great interest in forest management, as climate change is considered to be a major threat to forest health and sustainability [1]. Forest tree species of temperate and boreal regions have undergone a long history of demographic changes. During glacial maxima, many of these taxa were restricted to southern refugia, from where they expanded northwards during interglacials. Range contractions and expansions have been intensively studied using palaeobotanical and genetic approaches, demonstrating that past range changes were important determinants of the genetic structure of extant populations [2], [3], [4]. Genetic structures are likely to have been influenced also by evolutionary adaptations, enabling populations to adapt to local environments. In fact, provenance trials and genecological studies have revealedphenotypic traits with clear clines along diverse environmental gradients, both across species ranges and at the local scale [5], [6]. Yet, the underlying genes controlling adaptation remain poorly understood.

The development of forest tree genome sequences, single nucleotide polymorphisms (SNPs) databases and high-throughput genotyping platforms have facilitated the use of multi-locus scan approaches to identify loci involved in adaptation [7]. Two main groups of methods are currently used to identify loci related to adaptation. A first group is based on population differentiation and provides tools to detect loci that show significantly higher *F*_ST_ values than neutral expectations [8]–[11]. The second group of methods is based on correlations between allele frequencies and environmental variables and can be used to detect selection along gradients or in heterogeneous environments [12]. An important limitation of these methods is that they are sensitive to other evolutionary forces that can mimic selection, such as demographic history and population structure [11], [13].

Molecular studies in conifers, incorporating both population history and landscape features, have identified numerous loci, likely to be involved in adaptation [14]–[22]. These studies were mainly designed to investigate genetic diversity at the macro-geographic scale, i.e. across entire species ranges. Few studies have focused on a local scale, where gene flow is more effective and population structures are weak. On the other hand, gene flow can constrain adaptive divergence through homogenizing allele frequencies across space [23]. Nevertheless, when selection pressure is high, local adaptation may occur also at the local scale. For example, tree populations along altitudinal gradients often show pronounced clines in phenotypic traits [5], and thus may be well suited for detecting adaptive loci.

Norway spruce (*Picea abies* L. Karst.) is a broadly distributed European conifer of great ecological and economic importance. Its range is divided into two major regions, a northern, boreal region and a central and south-eastern European region [24]. In the southern region, Norway spruce mainly grows in mountains with widespread population occurrences found in the Alps, Carpathians, and Hercynia, the latter including the Bohemian massif and its surrounding mountains [24], [25]. The biogeography of Norway spruce has been intensively studied using fossil pollen [26] and genetic markers [27]–[31]. Surveys of genetic variation consistently revealed two distinct genetic lineages, separating populations of the north from those of the south [27], [28], [32]. Fossil pollen data combined with mitochondrial DNA data have shown that Norway spruce in the north is derived from a single large refugium, while in the south it persisted during the LGM in several distinct refugia [32]. At the phenotypic level, several potentially adaptive traits have been identified, such as bud set, bud burst [33], [34], and shoot growth [35], with clear geographic clines along latitudinal and altitudinal gradients. Notably, a recent study of northern populations using SNPs in functional genes has identified several components potentially involved in the control of bud set [19]. Other genes underlying local adaptation, however, remain unknown [36].

In this study, we focus on Norway spruce of central and south-easter Europe with the primary research goal of identifying adaptive loci through screening SNP markers at different geographic scales, taking into account population structures. SNP markers, representing 290 genes, were used to examine the role of genetic structure and environmental variation in shaping the distribution of species genetic variation and its adaptation. To achive this purpose, the sampling was designed at micro-geographic scale, where trees were sampled along two altitudinal gradients within the Alps and at macro-geographic scale, where trees were sampled in 27 natural populations across the southern range of Norway spruce. First, population structure was estimated to assess the possible presence of different genetic pools at micro- and macro-geographic scales. Second, to assess the role of selection in structuring genetic variation, we applied *F*_ST_-outlier methods taking into account the population structure, and tested for correlations between allele frequencies and climatic variables at both geographic scales.

## Materials and Methods

### Plant Material

Norway spruce is a very common and not endangered tree species in Europe. For each tree, approximately 500 mg of needle tissue was sampled. No specific permissions were required for these locations/activities and we did not sample in any protected areas. The geographic coordinates are reported in Table 1 and Table 2.

**Table 1:** Sampling sites included in the micro-geographic study area with their labels (Pop ID), provenances (Municipality), sample sizes (N), altitude (E), geographic coordinates (Lat: latitude; Long: longitude) and values of annual mean temperature (T) and precipitation (P).

Pop ID	Municipality	Lat (dec)	Long (dec)	N	E (m a.s.l.)	T(°C)	P(mm)	*H*_O_ mean	SD *H*_O_	*H*_E_ mean	SD *H*_E_	*F*_IS_	1-*Q*_inter_	monomorphic loci %
*South-western slope*														
C12	Celentino – Peio	46.20	10.43	26	1200	7.2	750	0.240	0.182	0.241	0.169	−0.0063	0.2490	11.89%
C14	Celentino – Peio	46.20	10.43	25	1400	6.2	738	0.259	0.195	0.244	0.171	0.0002	0.2487	11.01%
C16	Celentino – Peio	46.21	10.43	25	1600	5.1	730	0.254	0.194	0.244	0.175	0.0101	0.2484	10.57%
C18	Celentino – Peio	46.21	10.43	25	1800	4.0	716	0.251	0.189	0.244	0.172	0.0008	0.2496	8.37%
C20	Celentino – Peio	46.22	10.43	25	2000	2.9	712	0.254	0.182	0.246	0.164	0.0053	0.2501	6.17%
C22	Celentino – Peio	46.22	10.43	28	2200	1.7	704	0.247	0.183	0.244	0.173	−0.0233	0.2511	9.25%
*Northern slope*														
M10	Mezzana	46.18	10.48	25	1000	8.5	732	0.240	0.188	0.242	0.174	0.0196	0.2490	9.25%
M12	Mezzana	46.18	10.48	26	1200	7.4	747	0.252	0.177	0.247	0.167	−0.0266	0.2480	8.37%
M14	Mezzana	46.17	10.48	25	1400	6.3	771	0.248	0.181	0.244	0.170	−0.0079	0.2480	10.57%
M16	Mezzana	46.17	10.48	23	1600	5.5	791	0.248	0.185	0.243	0.171	0.0027	0.2510	8.81%
M18	Mezzana	46.16	10.47	23	1800	4.2	814	0.230	0.174	0.239	0.173	0.0206	0.2470	11.01%
M20	Mezzana	46.16	10.47	24	2000	3.2	847	0.252	0.187	0.241	0.170	−0.0248	0.2500	10.13%
								0.248	0.168	0.248	0.165			9.69%

Genetic variability across populations with mean values of observed (*H*_O_) and expected heterozygosity (*H*_E_) with its standard deviation (SD) and *F*_IS_ statistics per population over all loci with the gene diversity among individuals within population (1-Q_inter_).

**Table 2:** Sampling sites included in the macro-geographic investigation with their labels (Pop ID), provenances (Country), sample sizes (N), altitude (E), geographic coordinates (Lat: latitude; Long: longitude) and values of annual mean temperature (bio01) and precipitation (bio12).

Pop ID	Country	Lat (dec)	Long (dec)	N	bio01*	bio04*	bio09*	bio11*	bio12*	*H*_O_ mean	SD *H*_O_	*H*_E_ mean	SD *H*_E_	*F*_IS_	1-Q_inter_	monomorphic loci %
A1U	Austria	47.13	13.18	15	1.1	6156	−6.1	−6.8	1357	0.231	0.193	0.225	0.177	0.0022	0.231	18.57%
A2U	Austria	47.52	13.89	16	4.3	6775	−3.4	−4.7	1457	0.230	0.201	0.227	0.179	0.2346	0.230	18.99%
BOE	Switzerland	46.98	8.84	16	3.4	5681	−3.2	−3.7	1573	0.221	0.194	0.221	0.182	0.0401	0.224	23.63%
D2U	Germany	47.49	12.94	22	4.0	6084	−6.8	−7.4	1433	0.223	0.182	0.224	0.177	−0.0144	0.234	13.92%
M16	Italy	46.33	10.90	17	7.8	6451	−2.3	−2.3	750	0.219	0.192	0.220	0.180	−0.0053	0.231	17.72%
MN	Montenegro	42.70	20.10	15	5.6	6481	13.5	−2.8	1123	0.233	0.193	0.227	0.171	−0.1638	0.205	13.50%
POS	Switzerland	46.29	10.06	16	6.5	6396	−0.7	−1.7	875	0.222	0.192	0.223	0.181	0.0319	0.228	19.41%
S1U	Switzerland	46.03	7.10	24	3.3	6000	10.8	−4.3	1483	0.234	0.190	0.225	0.177	0.0260	0.228	16.88%
S3U	Switzerland	46.78	9.87	22	3.1	5811	−3.7	−4.3	1052	0.240	0.189	0.236	0.170	0.0293	0.212	10.13%
SBE	Switzerland	46.46	9.19	16	1.9	5537	−4.6	−4.9	1483	0.234	0.192	0.227	0.174	0.0105	0.218	17.30%
UA	Ukraine	48.12	24.46	21	3.0	7056	−5.5	−6.2	928	0.236	0.178	0.234	0.164	0.0109	0.243	8.44%
X1	Austria	46.50	14.60	23	6.3	7289	−1.9	−3.4	1154	0.215	0.198	0.216	0.179	0.0259	0.224	23.21%
X128	France	45.61	6.81	17	5.5	6276	13.2	−2.6	1227	0.239	0.253	0.200	0.191	0.0289	0.227	32.91%
X141	Germany	47.54	10.89	24	3.9	6079	−3.9	−3.9	1115	0.206	0.177	0.208	0.175	−0.0020	0.232	22.36%
X143	Germany	47.70	11.20	21	8.1	6670	0.6	−0.7	989	0.216	0.202	0.211	0.179	0.0515	0.234	24.05%
X168	Germany	50.70	10.70	21	5.9	6372	1.3	−2.3	879	0.240	0.186	0.237	0.173	0.0614	0.238	13.08%
X224	Poland	49.08	22.87	23	5.6	7507	−3.6	−4.6	821	0.222	0.182	0.229	0.176	0.0144	0.237	14.77%
X235	Poland	50.80	16.10	24	6.3	7306	−2.4	−3.7	688	0.224	0.173	0.233	0.172	0.0226	0.242	14.77%
X237	Poland	50.80	20.00	20	7.5	7822	−1.8	−3.4	634	0.234	0.182	0.233	0.167	0.0211	0.239	8.44%
X254	Romania	45.65	25.03	21	4.1	6900	−4.2	−5.2	867	0.237	0.177	0.238	0.172	−0.0015	0.243	8.86%
X258	Romania	46.66	25.77	22	4.8	7392	−4.1	−5.1	727	0.235	0.187	0.234	0.174	0.0250	0.234	14.35%
X267	Romania	47.70	25.60	15	5.5	7593	−3.6	−4.6	738	0.243	0.184	0.237	0.171	0.0497	0.238	9.28%
X29	Austria	47.52	15.03	20	2.3	6570	−5.2	−6.2	1267	0.238	0.196	0.229	0.174	−0.0112	0.230	13.50%
X301	Slovakia	49.30	20.50	23	5.1	7157	−3.5	−4.5	861	0.229	0.182	0.229	0.171	0.0022	0.241	14.77%
X304	Slovenia	45.58	14.45	24	5.7	6355	−1.7	−2.3	1344	0.227	0.193	0.230	0.180	−0.0160	0.230	19.41%
X350	Switzerland	46.48	6.96	24	4.0	6002	5.1	−3.6	1483	0.221	0.190	0.219	0.177	0.0104	0.224	15.61%
X63	CzechRep.	49.10	15.30	24	7.1	6929	−0.7	−2.0	714	0.232	0.198	0.224	0.179	0.2387	0.236	18.57%
										0.229	0.159	0.237	0.165			16.46%

Genetic variability across populations with mean values of observed (*H*_O_) and expected heterozygosity (*H*_E_) with its standard deviation (SD) and *F*_IS_ statistics per population over all loci with the gene diversity among individuals within population (1-Q_inter_).

* bio01  =  Annual Mean Temperature; bio04  =  Temperature Seasonality (standard deviation *100); bio09  =  Mean Temperature of Driest Quarter; bio11  =  Mean Temperature of Coldest Quarter; bio12  =  Annual Precipitation; Precipitation data is mm.

The micro-geographic scale study included two altitudinal transects on south-west (Celentino-Pejo) and north (Mezzana) aspects in the Trentino province (Italy) (Table 1). Six populations were sampled along each transect, with each of the populations separated by 200 m of altitude. On average, 25 adult trees (60–70 years old) were sampled from each site, for a total of 300 trees (Table 1).

The macro-geographic scale sampling consisted of 27 putatively natural populations, distributed across the range of Norway spruce in central- and south-eastern Europe. Each population was represented by 15–24 individuals, for a total of 546 trees. Eight of the populations were sampled in the IUFRO 1964/68 provenance test [37] (Table 2). To compare the micro- with the macro-geographic scale study, the Mezzana site located at 1600 m a.s.l. was included in the macro-geographic investigation and more sites were sampled in the Alps. Total DNA was extracted from needles according to Doyle and Doyle [38] or using the DNeasy 96 Plant Kit or the DNeasy Plant Mini Kit (QIAGEN, Hilden, Germany) according the manufacturer's instructions.

### Climatic data

In the micro-geographic scale study, two climatic variables were used. Ten years of average monthly mean temperature and monthly mean precipitation were obtained from the local spatial database [39] using climatic data collected from 1990 to 1999 by 64 weather-stations distributed in the Trentino province (Table 1).

In the macro-geographic scale study, we considered 19 bioclimatic variables, publicly available from the WorldClim - Global Climate Data (Free climate data for ecological modelling and GIS http://www.worldclim.org). Based on the species distribution and its ecological preferences, and to describe the sampling site climate, five bioclimatic variables were integrated in the analyses: mean annual temperature (bio01), temperature seasonality (bio04), mean temperature of the warmest quarter (bio09), mean temperature of the coldest quarter (bio11), and annual precipitation (bio12) (Table 2). Climatic data were collected from a 30 second GIS layer using Quantum GIS (Q-GIS) [40].

### SNP discovery and genotyping

SNP discovery was based on Sanger re-sequencing of a panel of 12 unrelated trees using primers derived from almost 1000 loblolly pine expressed sequence tags (ESTs), representing genes having various biological functions (http://dendrome.ucdavis.edu/NealeLab/crsp/overview.php). DNA was extracted from the haploid megagametophyte, obtained from one seed per sampled tree. Individual sequence alignment and SNP identification were performed using *PineSAP* [41]. A final set of 384 SNPs among those having quality design scores above 0.6, were selected for the genotyping, considering a maximum of two SNPs per locus, and preferring SNPs determining a change in predicted proteins (92 non-synonymous SNPs were selected). A total of 846 trees were genotyped. The SNP genotyping was performed at the Piattaforma Tecnologica Padana (Lodi, Italy) using the Illumina SNP bead array platform (Illumina, San Diego, USA) and the GoldenGate assay.

For each SNP, the percentage of individuals genotyped (call rate), the minor allele frequency (maf), the expected (*H*_E_) and observed (*H*_O_) heterozygosity, and the Wright's inbreeding coefficient (*F*_IS_) were calculated using the Genepop 4.0.5.3 program [42]. To remove uncertain and rare SNPs, loci with call rates <90%, maf <1%, or absolute *F*_IS_ values >0.25 were discarded. An individual call rate value was calculated for each sample, and samples with call rates lower than 95% were excluded.

### Genetic diversity

For each geographic scale, values of genetic diversity among individuals (*F*_IS_), among populations (*F*_ST_) and for the total population (*F*_IT_) were calculated for each locus using Genepop (S1B Supporting Material). With the same software, fixation index (*F*_IS_) statistics per population were calculated over all loci with the gene diversity among individuals within population (1-Q_inter_). Differences among populations were tested using the pairwise *F*_ST_ analysis in Arlequin 3.5 [43].

### *F*_ST_-outlier detection

To identify *F*_ST_ outliers, both Bayesian and coalescent simulations were applied. The first method considers individual locus effect and specific population, focusing on a genome scan for positive and balancing selection, as implemented in BayeScan 2.1 [11]. The method tests two alternative models and assigns a Bayes factor to each locus. We used a prior odd equal 10 and a false discovery rate FDR  = 0.001. The second method proposed by Excoffier [44] assumes two possible situations: an equal probability of migration between populations (finite island model) and the presence of structured populations (hierarchical island model). Both approaches were applied twice: using populations assigned according to their geographic position and according to STRUCTURE clustering.

### Regression analysis

To identify loci with extreme correlations between allele frequencies and climatic variables, regression analyses were carried out. We used linear regression models where the dependent variable was the arcsine-transformed major allele frequency (MAF) of each SNP, and the independent variables were climatic variables, ancestry coefficients, and an error term. The ancestry coefficients that describe the population structure were included as covariates, as suggested by Korves [45]. In the micro-geographic study, each SNP was tested in three models, considering the mean temperature, mean precipitation, and mean temperature and precipitation combined as independent variables (S1 Table). In the macro-geographic study, each SNP was tested in 9 models. Four models included a temperature variable (bio01, bio04, bio09, or bio11), one model the precipitation variable (bio12), and four models a temperature variable plus the precipitation variable (S1 Table). For each SNP, the model showing the minimum Akaike's Information Criterion (AIC) was selected as the best fit of the data. Using this model, the proportion of SNP variation explained by the climatic variables was estimated. FDR-corrected *P*-values (*Q*-values) were estimated using the software Q-value [46] implemented in R [47]. Correlations with *Q*-value <0.05 were considered as significant.

### Population structure

Patterns of population structure were analysed by principal component analysis (PCA) and by Bayesian cluster analysis. To further characterize population structures, hierarchical *F*-statistics and analysis of molecular variance (AMOVA) were applied [43]. The PCA was performed on the normalized genotypic data matrix. To identify the top *k* significant PCs, each PC eigenvalue was standardized and compared to the Tracy-Widom distribution (TW statistics) [48]. A significance cut-off of 5% was used to determine the significant PCs representing population structure. Then, hierarchical fixation indices were calculated from variance components according to Yang [49] as applied in the HIERFSTAT library [50] in R. Bayesian cluster analysis was performed on the SNP data matrix using the program STRUCTURE ver.2.2 [51] on Bioportal (www.bioportal.uio.no). STRUCTURE runs were performed with a Markov Chain Monte Carlo (MCMC) burn in of 500,000 steps, followed by an MCMC of 600,000 steps. An admixture model was used in the simulations. Each analysis was replicated 10 times for each *K*, with *K* ranging from 1 to 12 and from 1 to 30 at the micro- and macro-geographical scale, respectively. The best *K* was assigned using the log likelihood value, and populations were assigned to each genetic cluster considering the assignment of the majority of individuals within each population.

To investigate partitioning of genetic variation at different hierarchical levels, and to corroborate the results obtained with HIERFSTAT and STRUCTURE, an AMOVA analysis was performed at both geographic scales, assuming the presence of four genetic groups (see Results) at the macro-geographic scale, and two groups (transects) at the micro-geographic scale. The AMOVA was performed using Arlequin software [43].

## Results

The 384 SNPs considered represent 290 genes (S1A Supporting Material) encoding proteins with various biological functions. Among those SNPs, 41 failed to amplify, 63 were monomorphic in all samples, and 54 SNPs (micro-geographic scale) and 43 (macro-geographic scale) did not pass the quality control. A total of 226 SNPs across 224 genes (micro-geographic scale) and 237 SNPs across 247 genes (macro-geographic scale) were successfully genotyped.

### Genetic diversity

At the micro-geographic scale, the overall genetic diversity (considering all SNPs together) expressed as observed heterzygosity (*H*_O_) per population ranged from 0.230 (M18) to 0.259 (C14) with a grand mean of 0.248 (± SD  = 0.168) (Table 1). At the macro-geographic scale (Table 2), *H*_O_ was between 0.206 (population X141) and 0.243 (X267) with a grand mean of 0.229 (± SD  = 0.159).

No significant differences were found in *F*_ST_ value between population pairs at the micro-geographic scale (S2A Table). At the macro-geographic scale, several population pairs had a significant *F*_ST_-value (*P*<0.0001) according to the permutation test (S2B Table) and the *F*_ST_ values were between 0.012 (X350 and S1U) and 0.680 (BOE and MN).

### *F*_ST_-outliers detection

*F*_ST_ values were calculated for each locus at both geographic scales following Weir and Cockerham [52]. At the micro-geographic scale, no outliers were detected using either the Bayesian simulation or considering the neutral island model.

At the macro-geographic scale, *F_S_*_T_ *-*values calculated among populations varied between -0.008 and 0.36 (S1B Supporting Material; mean *F*_ST_ = 0.024). The BayeScan simulation was run twice: using populations assigned according to their geographic position and according to STRUCTURE clustering. The first simulation detected 8 outlier loci (Table 3 and S1A Fig.). The outlier with the highest *F*_ST_-value (0.234), SNP locus 2_10483_01-340, encodes a haloacid dehalogenase-like hydrolase and was detected only in Alpine populations (S2 Fig.). The other SNP loci encode a sucrose synthase (CL813Contig1_03), a transcription factor (0_10267_01), translation-elongation factor (0_8642_01), UBX domain-containing protein (0_9922_01), acyl-CoA thioesterase (2_8491_01), acetyltransferase component (CL866Contig1_01) and an unknown protein (2_5073_01-321). BayeScan simulations taking into account the four STRUCTURE clusters identified a single outlier (1_3086_01-101; *F*_ST_ = 0.128; S1B Fig.) that was not detected in the first simulation (S1A Fig.). This locus encodes a protein of unknown function (Table 3).

**Table 3:** Outlier detection using BayeScan results at the macro-geographic scale: populations assigned according their geographic position (*All populations*), according to all STRUCTURE clusters (*all-clusters*).

SNP	Putative function	*F*_ST_	*All populations*				*STRUCTURE all-clusters*				*Island model*			*Hierarchical island model*	
			log_10_ PO	prob	*Q*val	alpha	log_10_ PO	prob	*q*val	alpha	*He*_obs_	*F*_ST obs_	*F*_ST_ *P*-value	*F*_CT obs_	*F*_CT_ *P*-value
0_10267_01-274	R2R3-MYB transcription factor MYB8	0.0235	0.846	0.875	0.049	−1.091									
0_8642_01-166	translation elongation factor EF-G	0.1298	1.159	0.935	0.033	1.061									
0_9922_01-345	UBX domain-containing protein	0.1309	1.135	0.932	0.038	1.071									
2_10483_01-340	haloacid dehalogenase-like	0.2336	1.000	1.000	0.000	1.994					0.272	0.407	0.000	0.200	0.000
2_5073_01-321	Unknown	0.0183	1.307	0.953	0.022	−1.373									
2_8491_01-519	acyl-CoA thioesterase, putative	0.1304	1.295	0.952	0.027	1.073									
CL813Contig1_03-235	sucrose synthase	0.1585	0.999	2.743	0.001	1.367					0.118	0.138	0.000	0.128	0.000
CL866Contig1_01-360	acetyltransferase component of pyruvate dehydrogenase	0.0183	1.398	0.961	0.013	−1.346									
1_3086_01-101	NA	0.1277					1.289	0.951	0.048	1.486	0.178	0.133	0.000	0.122	0.000
0_12021_01-161	ovule receptor-like kinase 28	0.0757									0.260	0.161	0.000	0.156	0.000
CL4578Contig1_02-154	NA	0.0545									0.213	0.118	0.000	0.107	0.000
UMN_4091_02-458	F-box family protein	0.0544												0.000	0.000

Outlier detected using Arlequin with the neutral island and hierarchical island model assumptions; only loci highly significant (*P*<0.0001) are reported in the table.

Among the outliers detected by coalescent simulations assuming a neutral island model, five were highly significant (*P*<0.0001; Table 3). Three of them (2_10483_01-340, CL813Contig1_03-235, 1_3086_01-101) overlapped with those identified by BayeScan. The other two encode an ovule receptor-like kinase (0_12021_01) and a protein with unknown function (CL4578Contig1_02). Simulations considering the four STRUCTURE clusters identified the same five outliers and an additional outlier (UMN_4091_02-458), with its locus encoding an F-box family protein.

### Environmental associations

Associations between allele frequencies and climatic variables were analysed with linear regression models taking into account ancestry coefficients. At the micro-geographic scale, the 227 SNPs were analysed with three models, including either temperature, precipitation, or temperature and precipitation combined. Overall, two SNP loci showed a significant correlation with climate (Table 4; S2A Supporting Material). One of them (2_5636_01), encoding a pentatricopeptide repeat-containing protein, was correlated with annual mean temperature, and the other (2_9466_01-179) with mean temperature and precipitation combined. The latter locus was shared with the macro-geographic scale study. The amount of its frequency variation explained by climate was 83%.

**Table 4:** Summary of significant regression models according to the FDR (False Discovery Rate) method [66].

Locus		Putative function	model	*P*	*R*	*P*_var1_	*P*_var2_
*Micro-geographic scale*							
**2_2960_02-335**	NA		M3	0.0004	0.8254	0.0005	0.0044
**2_5636_01-399**	pentatricopeptide (PPR) repeat-containing protein		M1	0.0017	0.6447	0.0017	NS
					
*Macro-geographic scale*							
**0_13913_02-313**	exocyst subunit EXO70 family proteinG1		M1	0.0005	0.469	0.0063	NA
0_17215_01-108	magnesium chelatase H-like protein		M1	0.0030	0.384	0.4669	NA
0_7844_01-303	vernalization insensitive 3		M1	0.0000	0.5767	0.4052	NA
0_9922_01-345	UBX domain-containing protein		M1	0.0000	0.5760	0.2303	NA
2_2937_01-127	unknown protein		M1	0.0025	0.3931	0.0007	NA
2_4029_01-212	receptor protein kinase, putative		M1	0.0012	0.4308	0.0325	NA
2_6491_01-360	unknown protein		M1	0.0001	0.5291	0.1651	NA
2_8491_01-519	acyl-CoA thioesterase, putative		M1	0.0021	0.4025	0.2968	0.1965
CL71Contig1_04-119	disease resistance associated protein		M1	0.0000	0.6668	0.6560	NA
**0_1688_02-505**	ATP binding protein, putative		M2	0.0023	0.3971	0.0009	NA
**0_7471_01-132**	NA		M2	0.0019	0.4063	0.0087	NA
2_8852_01-381	galactokinase, putative		M2	0.0020	0.4049	0.0244	NA
**CL3363Contig1_04-85**	GTP binding		M2	0.0037	0.3732	0.0050	NA
**UMN_1787_01-240**	NA		M2	0.0008	0.4473	0.1130	0.0021
**0_5583_01-181**	hypothetical protein		M4	0.0005	0.4697	0.0036	NA
CL1688Contig1_01-463	beta-D-galactosidase		M4	0.0001	0.5524	0.0294	NA
CL3602Contig1_03-56	NADPH		M4	0.0004	0.4840	0.0497	NA
CL3795Contig1_01-45	C-1-tetrahydrofolate synthase		M4	0.0009	0.4401	0.0203	0.0967
0_10910_02-321	unknown protein		M5	0.0003	0.4947	0.0065	0.0489
0_12021_01-161	ovule receptor-like kinase 28		M5	0.0001	0.5525	0.0482	NA
0_13552_02-284	hypothetical protein		M5	0.0001	0.5272	0.3838	NA
0_2643_01-338	NA		M5	0.0019	0.4070	0.0669	NA
0_8642_01-166	translation elongation factor EF-G		M5	0.0003	0.4984	0.4477	NA
**2_2960_02-335**	NA		M5	0.0019	0.4078	0.0112	NA
2_3591_03-327	hypothetical protein		M5	0.0001	0.5217	0.0133	NA
2_4281_02-310	subtilase family protein		M5	0.0024	0.3957	0.3705	NA
2_8852_01-97	galactokinase, putative		M5	0.0000	0.6402	0.0114	NA
2_9466_01-179	membrane-associated zinc protease		M5	0.0000	0.7174	0.0007	NA
CL4511Contig1_02-223	oligopeptidase, putative		M5	0.0001	0.5304	0.0894	NA
UMN_1908_01-593	adaptin family protein		M5	0.0007	0.4522	0.0003	NA
2_6355_02-53	NA		M6	0.0001	0.5562	0.1555	NA
CL304Contig1_01-118	oxygen-evolving complex protein 1		M6	0.0005	0.4714	0.0450	NA
**2_3867_02-440**	profilin		M7	0.0018	0.4732	0.3085	0.0035
0_4829_01-288	Aldose 1-epimerase family protein, expressed		M8	0.0002	0.5589	0.1058	NA
2_10483_01-340	haloacid dehalogenase-like hydrolase domain-containing protein 1A		M8	0.0001	0.5824	0.0278	NA
**2_3851_01-280**	unknown protein		M8	0.0006	0.5219	0.0048	0.1101
CL813Contig1_03-235	sucrose synthase		M8	0	0.6415	0.0388	
CL3862Contig1_06-366	mitogen-activated protein kinase 4		M9	0.0008	0.5105	0.0690	NA

*P* is the test probability for the selected model, *P*_var_ is the variable (var) probability, *R* is the linear correlation coefficient. NS: not significant, NA = not present. Loci with a *P*_var_<0.01 are in bold.

For each of the 237 SNPs used in the macro-geographic scale study, 9 models were applied, which included one or two climatic variables related to temperature or precipitation (S1 Table). The analyses resulted in the identification of 38 SNPs significantly correlated (at *Q*<0.05) with either temperature, precipitation, or temperature and precipitation variables combined (Table 4, S2B Supporting Material). Twelve loci were significant determinants (*P*_var_<0.01) in the model. The amount of SNP frequency variation explained by climatic variables ranged from 37% (CL3363Contig1_04-85) to 72% (2_9466_01-179), the latter locus encoding a hypothetical protein. Six of the identified loci overlapped with *F*_ST_-outliers (0_9922_01-345, 2_8491_01-519, 0_12021_01-161, 0_8642_01-166, 2_10483_01-340, CL813Contig1_03-235).

### Population structure

**Figure 1:**
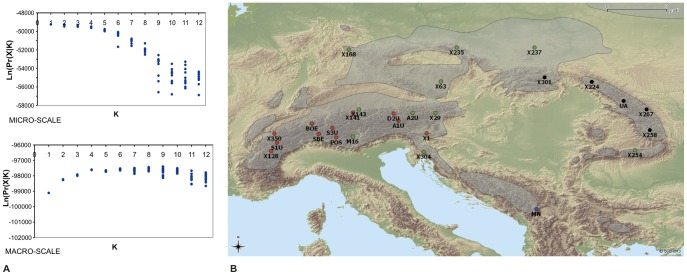
Bayesian cluster analysis using STRUCTURE [51]. Log likelihood value (Ln(Pr(X|*K*)) of Pritchard plot is shown for micro and macro-geographic scales(**A**). Macro-geographic populations clustering according to the Bayesian method implemented in STRUCTURE (B). The population dot colours represent the cluster that includes the majority of individuals within populations. The species distribution range is in green (created using Q-GIS based on description from [25]).

To examine patterns of population structure, PCA and Bayesian clustering were applied at both geographic scales. At the micro-geographic scale, PCA identified only one significant PC according to the TW statistics. The absence of population stratification was confirmed by HIERFSTAT and STRUCTURE analyses. The average level of genetic differentiation among sampling sites was extremely low, both between transects (*F*_transect/total_ = 0.0002, *P* = 0.587) and between sampling sites within transects (*F*_sampling_site/transect_ = 0.0018, *P* = 1) (S3A Table), and no clusters were identified using STRUCTURE (Fig. 1A). A further confirmation of the lack of structure at the micro-geographic scale was provided by the AMOVA (S3B Table).

At the macro-geographic scale, the PCA showed a significant population structure (S3A Fig.). Three PCs, explaining 13.1% of the total variance, were significant at the 5% threshold. The first PC was significantly correlated with longitude (*r^2^* = 0.11; *P* = 1.9e^−15^) and distinguished between populations of the Alps from all other populations. The second PC highlighted the peculiarity of a population (MN) located in Montenegro in the Dinaric Alps; it represented the southernmost population included in the study, and explained the correlation between PC2 and latitude (*R^2^* = 0.12, *P*<2.2e^−16^). The Bayesian cluster analysis with STRUCTURE detected four clusters (Fig. 1B). The population structure was similar to that of the PCA, but the populations of the Carpathians were separated from those of Hercynia. All but one of the populations of the Carpathians formed a first cluster. The second cluster included 11 of the 16 populations of the Alps. The third cluster was characterised by populations of Hercynia and included the five remaining populations of the Alps. As the PCA, Bayesian clustering assigned the Montenegro population to a separate cluster. To further characterize the population clustering, each population was assigned to the cluster that includes the majority of the samples, and the percent of variation among the four clusters was calculated with AMOVA (S3B Table). The analysis revealed that a very low (1.54%), but highly significant (*P*<0.0001) portion of the total variation is explained by differences among clusters, as confirmed by the *F*-statistics analysis (S3A Table).

## Discussion

This research confirms the findings from previous studies describing the genetic structure of Norway spruce at the European level and highlights the importance of integrating the effects of demography in outlier detection studies. The experimental design we used (micro- and macro-geographic scale) and the application of different approaches in the data analysis provided new insights into the underlying genes that may be responsible for local adaptation. Some potential adaptive loci were found to be associated to temperature at both geographic scales, confirming the importance of this factor in driving adaptation in forest species.

### Signature of adaptation

Theoretical [53] and empirical [17] studies show that the demographic history can inflate the detection of *F*_ST_ outliers. In the macro-geographic scale study, 13% of the variance was explained by population structure, presumably due to population demographic changes. Consequently, both BayeScan and Arlequin simulations were carried out with and without taking into account the population structure. BayeScan simulations without population structure correction detected eight outliers, whereas only one locus was detected with structure correction. The simulation considering the hierarchical island model detected seven outlier loci, with two of them being found only with this method. Altogether, seven *F*_ST_ outliers were identified taking into account the population structure, corresponding to 2.95% of the SNPs tested. This discovery rate is comparable to that observed in other conifers using similar approaches [14], [16], [18], including black spruce (*Picea mariana*), where few SNPs were identified as outliers, and only within a specific lineage [21].

To account for variation along clines due to demographic processes and/or selection, population structure was included as a covariate in the linear regression models. In the 27 populations analysed in the macro-geographic scale study, 38 SNP loci were significantly correlated with temperature and/or precipitation variables, corresponding to a 16% discovery rate. A greater ratio (22%) was detected in loblolly pine (*Pinus taeda*) using a different model [16]. The majority of these SNP loci showed significant correlations in models with temperature variables, consistent with results observed in a lodgepole pine (*Pinus contorta* Dougl. ex Loud) field transplant experiment [54] and in other coniferous species [6], suggesting that temperature is a significant force in shaping genetic diversity.

In the micro-geographic scale study, the number of loci potentially involved in adaptation was much smaller: no *F*_ST_-outliers were detected and the regression analyses identified only two SNPs significantly correlated with climatic variables. This finding was unexpected, because Alpine slopes are highly variable environments, where small changes in altitude can lead to significant variation in temperature, humidity and soil composition [55]. Adaptation of populations to such environments is likely to result in genetic clines associated with altitude. The average differentiation between populations distributed along the two altitudinal transects was very low, comparable to that previously reported for Norway spruce at the local scale [56], and is in accordance with estimates of gene flow described for tree-line ecotones [57]. This low differentiation was confirmed by Bayesian and PCA clustering, which both revealed absence of population structure. Assuming high levels of gene flow, it seems likely that gene flow constrained the effects of selection, at least to some extent. On the other hand, Norway spruce populations growing along altitudinal gradients typically show clear clines in growth and timing of bud set, indicating strong diversifying selection [19]. In our study, we tested only a limited number of loci, whose selection was largely based on quality scores derived from the original sequence data, rather than on functional annotations. Identification of loci involved in growth and bud set control would require analysis of more genes.

The higher rate of locus discovery at the macro- than at the micro-geographical scale may also be a result of spatial heterogeneity in selection regimes. In particular, the Alps, Carpathians and the Bohemian massif differ considerably in topography and their continental location, and thus are characterised by distinct climatic conditions. Notably, quantitative traits assessed in provenance trials revealed clear differences among populations of the Alps, Carpathians, and Hercynia, supporting different selection regimes for these areas [58]. It is therefore likely, that heterogeneity in selection regimes contributed to the signatures of selection we identified at the macro-geographic scale.

### Putative adaptive SNPs

The six SNP loci that were identified by both correlation-based and *F*_ST_-outlier analyses were considered as ‘putative adaptive loci’. The locus with the highest *F_ST_* value (2_10483_01) encodes a haloacid dehaolgenase-like hydrolase, an enzyme with a putative function in the biosynthetic pathway of the vitamin riboflavin, playing a role in a variety of redox processes affected in plant defence responses [59]. The SNP was significantly correlated with combined temperature and precipitation, and was only found in a subset of Alpine populations, further supporting its potential role in adaptation. At the micro-geographic scale, the frequency of its particular allele was very low, possibly explaining why this locus was not detected as an *F*_ST_-outlier. An additional candidate locus (CL813Contig1_03) encodes a sucrose synthase, an enzyme of the primary metabolism and responsible for energy supply. The expressed sequence tag was isolated from Aleppo pine (*Pinus halepensis*) and was shown to be induced by water stress [60], consistent with our finding, that the SNP was correlated with annual precipitation. Allelic changes in enyzmes of the primary metabolism are a general response of plants to stress [61], and in the case of sucrose synthase a function in water stress tolerance has been proposed [62]. A third locus (2_8491_01) encodes an acyl-CoA thioesterase and was associated to annual mean temperature. This enzyme catalyses the hydrolysis of acyl-CoAs to free-fatty-acid and coenzyme A, and thus regulates the intracellular levels of acyl-CoAs and free-fatty-acids. In white spruce (*Picea glauca*), acetoacetyl-CoA thiolase was demonstrated to be involved in the up-regulation of transcripts in response to stress [63]. The remaining three loci were a translation elongation factor, an UBX domain-containing protein, and an ovule receptor-like kinase protein.

### Population structure

Since genetic structure was detected only at macro-geographic scale, we assumed that both altitudinal transects sampled at micro-geographic scale belonged to the same gene pool. No structure effects at micro-geographic scale were observed.

At the macrogeographic scale, the analysis of population structure using SNPs of functional genes revealed three major clusters, which were largely congruent with those delineated in previous studies [32]. The most detailed information about the glacial and postglacial history of Norway spruce has been provided by the combined analysis of fossil pollen and mitochondrial DNA [26], [32]. The data indicate that Norway spruce of the southern part expanded out of three major refugia, giving rise to populations in the Alps, Hercynia, and the Carpathians. The cluster with Alpine populations, identified by both PCA and Bayesian clustering, corresponds to a mitochondrial lineage derived from a refugium probably located in the south-eastern Alps. Populations of Hercynia and the Carpathians were delineated only by Bayesian clustering, and probably corresponds to mitochondrial lineages derived from refugia located in the southern Bohemian massif and Carpathians. Both PCA and Bayesian clustering assigned the Montenegro population to a separate cluster. Compared to other populations, its population differentiation was quite high, which may be a result of a distinct glacial history and/or its occurrence at the southern range limit. Norway spruce in the southern Dinaric Alps typically occurs in scattered populations [24], which may promote genetic drift and thus population differentiation.

In this study, we confirmed the confounding effect of genetic structure in the detection of outlier loci (see previous section). Therefore, the estimation of species genetic structure is a crucial step in the identification of adaptive loci, as previously reported [16]; [19]; [21].

## Conclusions

This study indicates that genetic diversity of Norway spruce was shaped by both demographic and evolutionary processes, confirming the population structure identified with other marker types, but inferred from a much lower number of loci. The structure results were taken into account in the detection of selection and adaptation signs at the molecular level. The combined analyses of *F*_ST_-outliers and environmental associations led to the identification of several potential adaptive genes and corroborates previous suggestions that temperature is an important factor in shaping genetic diversity in conifers. A strong relation was found between genetic structure and environmental variables but this correlation does not allow the identification of the physiological function affected by the environmental factor. Therefore, in future studies it is crucial to complement genetic studies with transplant experiments, where the phenotypic variation or the effect of an environmental stress could be assessed. Finally, our original aspect of sampling at different spatial scales allowed us to provide insights into the effects of gene flow on local adaptation. Moreover, our results highlighted the importance of combining different approaches to investigate species adaptation [64], [65].

## Supporting Information

S1 Fig **BayeScan results at macro-geographic scale: populations assigned according to their geographic position (A) and according to STRUCTURE clustering (B).**
(TIFF)

S2 Fig **Plots of some loci significantly associated with bioclimatic variables; colours identify the locus minor allele frequency (m.a.f.) within each population.**
(TIFF)

S3 Fig **Plot of the first two significant principal components (PCs) at micro-geographic scale: one cluster was identified.** (**A**). Plot of the two first PCs at the macro-geographic scale (B). Population labels are coloured according to the populations ID. Eingvalues for all PCs are in the bar plots.
(TIFF)

S1 Table **Models used for the regression analysis at the micro- and the macro-geographic scale.** The letter “A” represents the population structure. The following variables were used: major allele frequency (MAF) with the arcsin transformation (asin(MAF)), annual mean temperature (T, bio01), annual precipitation (P, bio12), temperature seasonality (bio04), mean temperature of driest quarter (bio09) and mean temperature of coldest quarter (bio11).
(DOC)

S2 Table **Pairwise** ***F*** **_ST_ between population-pairs at micro- (A) and macro-geographic scale (B).** Population ID is described in Table 1 and Table 2. Values in bold are significantly different (*P*-value <0.0001) according to a permutation test (N = 1000).
(DOC)

S3 Table **Analysis of variance at micro- and macro-geographic scales.** *F* statistics were calculated at different levels using HIERFSTAT library in R: between transects or among clusters, among populations, among samples (A). AMOVA analysis calculated using Arlequin at micro- and macro-geographic scales. Fixation indexes statistically significant (*** *P*<0.000) (B).
(DOC)

S1 Supporting Material **SNP position and locus ID with BLAST-N (A).** *F*_IT_, *F*_ST_ and *F*_IS_ values calculated per locus at both scales (B). (XLS)
(XLS)

S2 Supporting Material **Regression model analysis at micro- (A) and macro-geographic scale (B).**
(XLS)

S3 Supporting Material **Sample by SNP genotyping matrix for micro- (A) and macro-geographic (B) studies.**
(XLS)
