# Supplementary figures and images for: Micro- and Macro-Geographic Scale Effect on the Molecular Imprint of Selection and Adaptation in Norway Spruce
Source: PLoS One. 2014 Dec 31;9(12):e115499. doi: 10.1371/journal.pone.0115499 (PMC4281139; doi:10.1371/journal.pone.0115499)

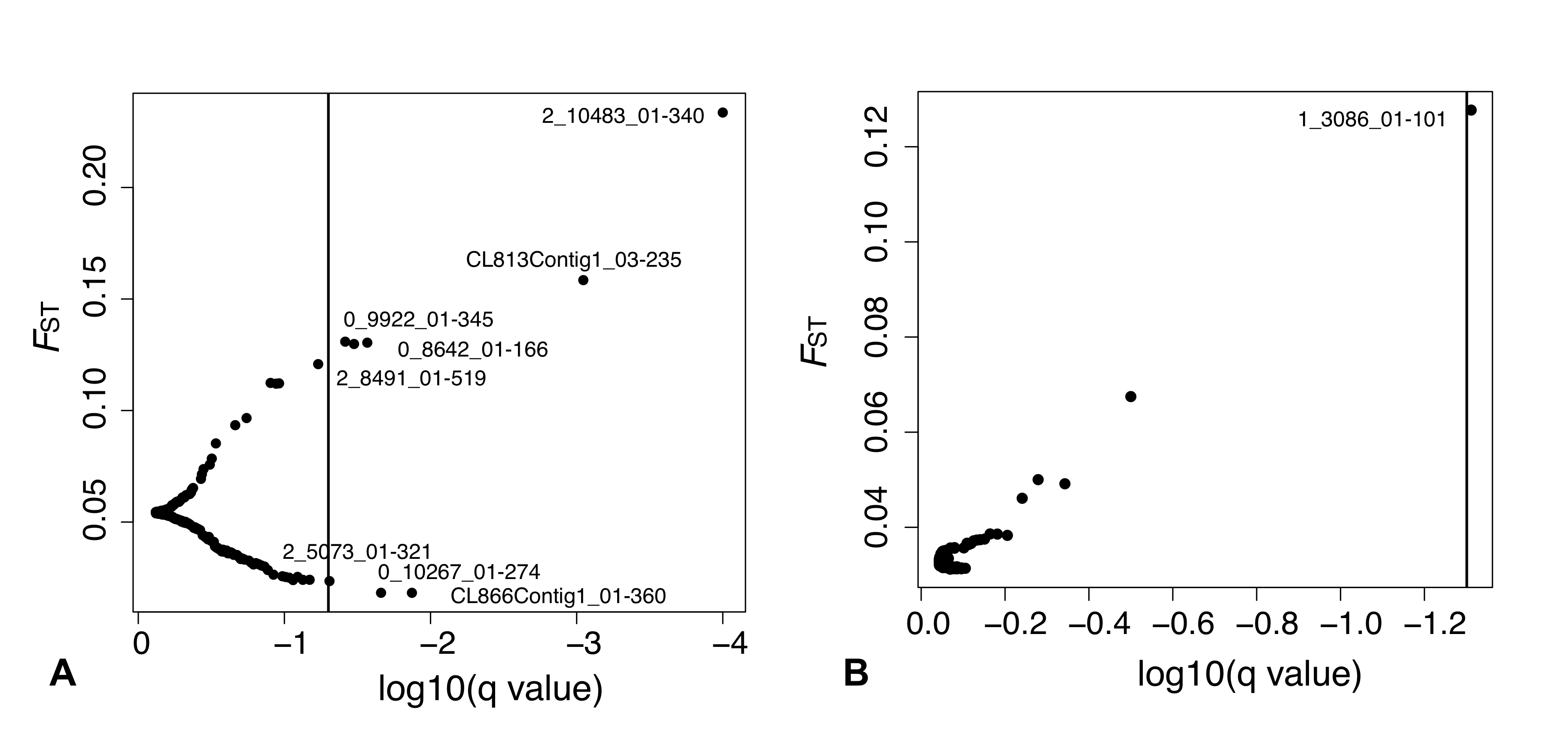

Supplement: S1 Fig — BayeScan results at macro-geographic scale: populations assigned according to their geographic position (A) and according to STRUCTURE clustering (B). (TIFF) [file pone.0115499.s001.tiff]

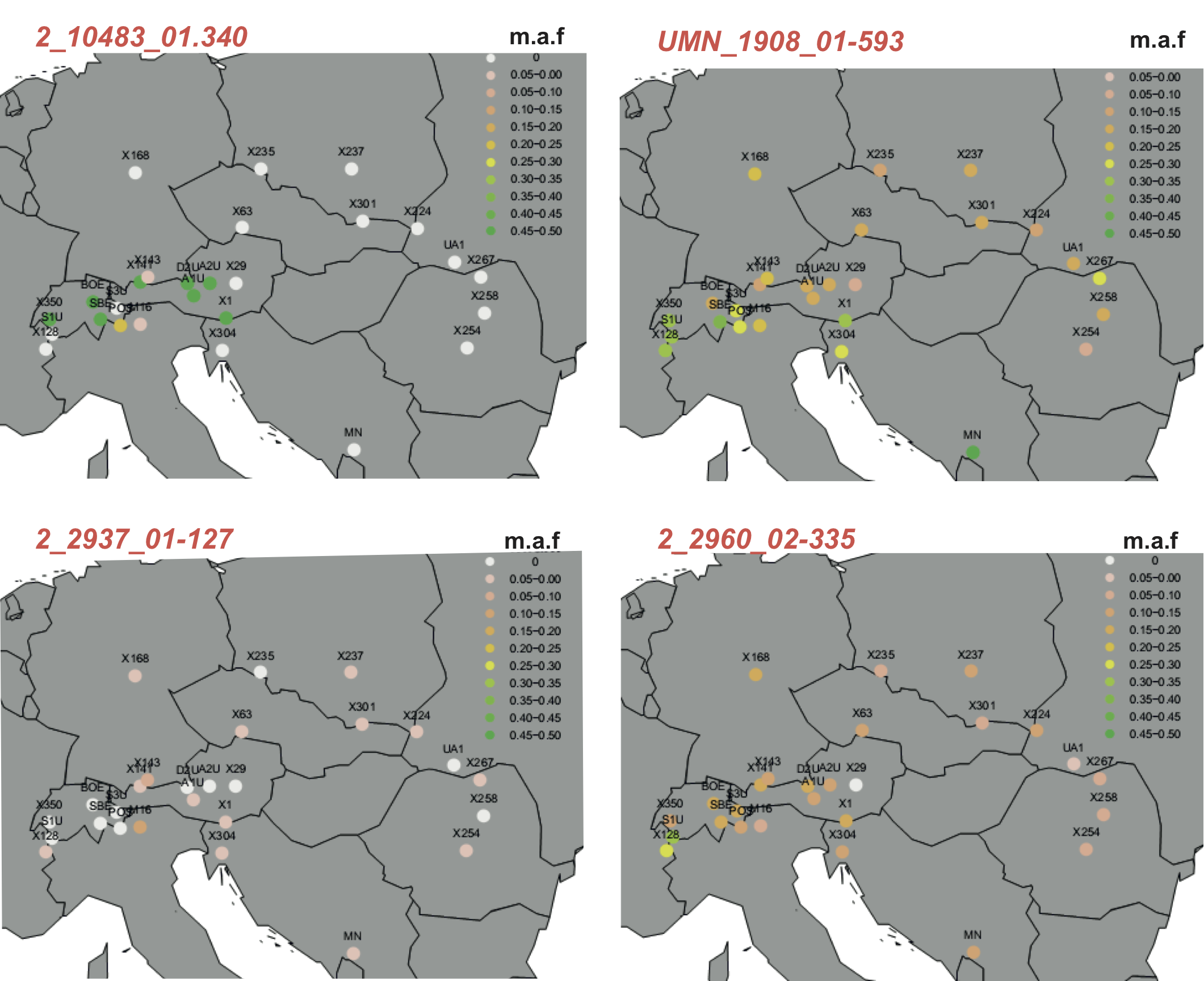

Supplement: S2 Fig — Plots of some loci significantly associated with bioclimatic variables; colours identify the locus minor allele frequency (m.a.f.) within each population. (TIFF) [file pone.0115499.s002.tiff]

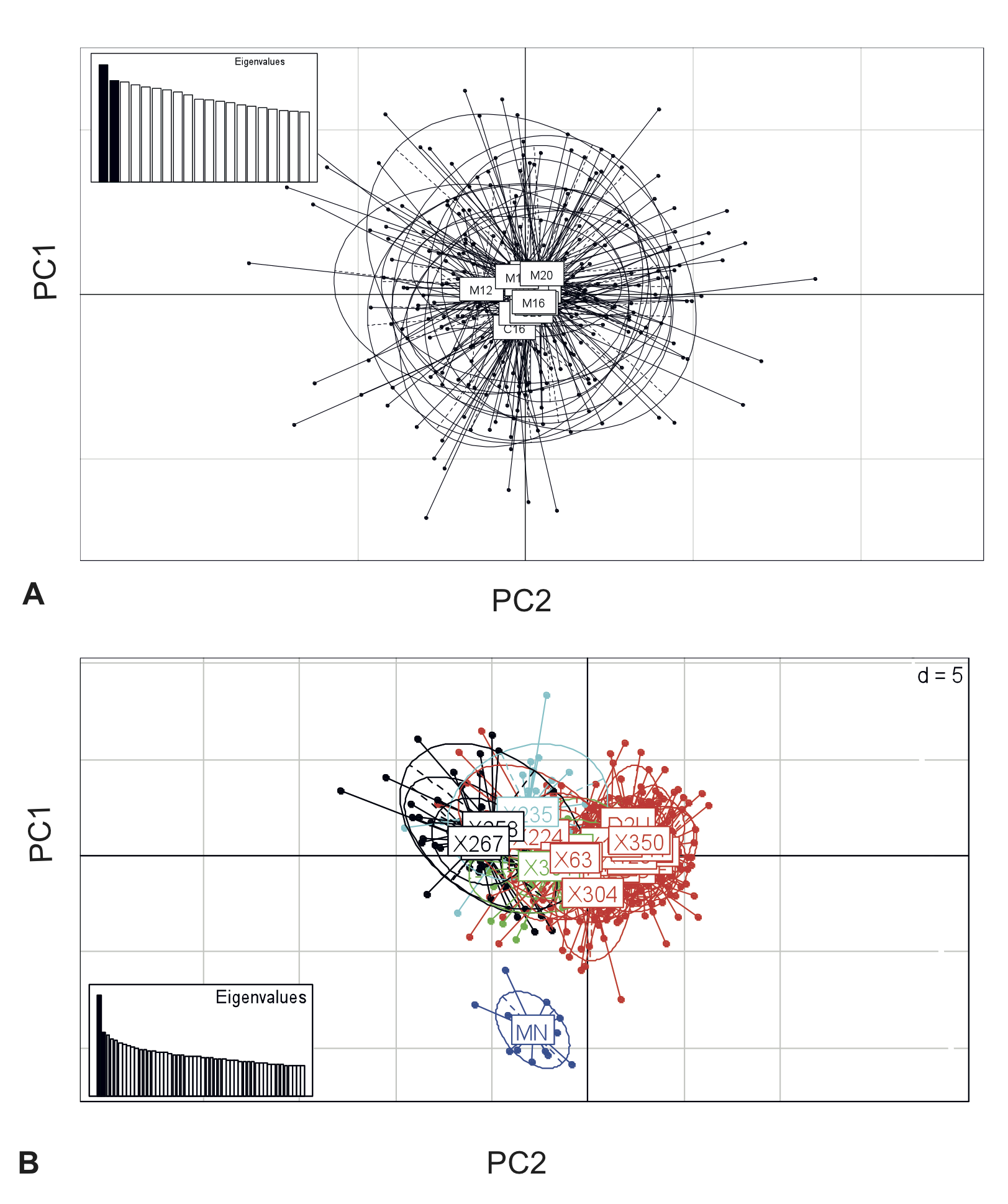

Supplement: S3 Fig — Plot of the first two significant principal components (PCs) at micro-geographic scale: one cluster was identified. (A). Plot of the two first PCs at the macro-geographic scale (B). Population labels are coloured according to the populations ID. Eingvalues for all PCs are in the bar plots. (TIFF) [file pone.0115499.s003.tiff]
