# Supplementary material for: Micro- and Macro-Geographic Scale Effect on the Molecular Imprint of Selection and Adaptation in Norway Spruce
Source: PLoS One. 2014 Dec 31;9(12):e115499. doi: 10.1371/journal.pone.0115499 (PMC4281139; doi:10.1371/journal.pone.0115499)
Supplement: S1 Table — Models used for the regression analysis at the micro- and the macro-geographic scale. The letter “A” represents the population structure. The following variables were used: major allele frequency (MAF) with the arcsin transformation (asin(MAF)), annual mean temperature (T, bio01), annual precipitation (P, bio12), temperature seasonality (bio04), mean temperature of driest quarter (bio09) and mean temperature of coldest quarter (bio11). (DOC) [file pone.0115499.s004.doc]

**Table S1:** Models used for the regression analysis at the micro- and the macro-geographic scales. The letter “A” represents the population structure. The following variables were used: major allele frequency (MAF) with the arcsin transformation (asin(MAF)), annual mean temperature (T, bio01), annual precipitation (P, bio12), temperature seasonality (bio04), mean temperature of driest quarter (bio09) and mean temperature of coldest quarter (bio11).

| **Investigation Scale** | **Model** |
| --- | --- |
| Micro |  |
|  | Model_1 (m1): asin(MAF) ~ T + ε |
|  | Model_2 (m2): asin(MAF) ~ P + ε |
|  | Model_3 (m3): asin(MAF) ~ P + T + ε |
|  |  |
| Macro | Model_1 (m1): asin(MAF) ~ A+bio01+ ε |
|  | Model_2 (m2): asin(MAF) ~ A+bio12+ ε |
|  | Model_3(m3): asin(MAF) ~ A+ bio12+ bio01+ ε |
|  | Model_4 (m4): asin(MAF) ~ A+bio04+ ε |
|  | Model_5 (m5): asin(MAF) ~ A+bio09+ ε |
|  | Model_6 (m6): asin(MAF) ~ A+bio11+ ε |
|  | Model_7 (m7): asin(MAF) ~ A+ bio12+ bio04+ ε |
|  | Model_8 (m8): asin(MAF) ~ A+ bio12+ bio09+ ε |
|  | Model_9 (m9): asin(MAF) ~ A+ bio12+ bio11+ ε |
|  |  |
|  |  |
|  |  |
|  |  |
|  |  |
