# Supplementary material for: Micro- and Macro-Geographic Scale Effect on the Molecular Imprint of Selection and Adaptation in Norway Spruce
Source: PLoS One. 2014 Dec 31;9(12):e115499. doi: 10.1371/journal.pone.0115499 (PMC4281139; doi:10.1371/journal.pone.0115499)
Supplement: S2 Table — Pairwise F ST between population-pairs at micro- (A) and macro-geographic scale (B). Population ID is described in Table 1 and Table 2. Values in bold are significantly different (P-value <0.0001) according to a permutation test (N = 1000). (DOC) [file pone.0115499.s005.doc]

**Table S2**: Pairwise *F*_ST_ between population-pairs at micro- (A) and macro-geographic scale (B). Population ID is described in Table 1. Values in bold are significantly different (*P*-value<0.0001) according to a permutation test (N=1000).

A

|  | 1 | 2 | 3 | 4 | 5 | 6 | 7 | 8 | 9 | 10 | 11 | 12 |
| --- | --- | --- | --- | --- | --- | --- | --- | --- | --- | --- | --- | --- |
| 1 | 0.000 |  |  |  |  |  |  |  |  |  |  |  |
| 2 | -0.010 | 0.000 |  |  |  |  |  |  |  |  |  |  |
| 3 | -0.007 | -0.012 | 0.000 |  |  |  |  |  |  |  |  |  |
| 4 | -0.012 | -0.010 | -0.011 | 0.000 |  |  |  |  |  |  |  |  |
| 5 | -0.011 | -0.014 | -0.014 | -0.013 | 0.000 |  |  |  |  |  |  |  |
| 6 | -0.010 | -0.009 | -0.011 | -0.013 | -0.012 | 0.000 |  |  |  |  |  |  |
| 7 | -0.008 | -0.010 | -0.013 | -0.012 | -0.015 | -0.012 | 0.000 |  |  |  |  |  |
| 8 | -0.009 | -0.015 | -0.014 | -0.011 | -0.014 | -0.011 | -0.013 | 0.000 |  |  |  |  |
| 9 | -0.008 | -0.014 | -0.012 | -0.011 | -0.014 | -0.011 | -0.012 | -0.012 | 0.000 |  |  |  |
| 10 | -0.012 | -0.015 | -0.012 | -0.012 | -0.015 | -0.011 | -0.012 | -0.015 | -0.013 | 0.000 |  |  |
| 11 | -0.008 | -0.013 | -0.010 | -0.010 | -0.011 | -0.012 | -0.011 | -0.014 | -0.016 | -0.014 | 0.000 |  |
| 12 | -0.012 | -0.012 | -0.012 | -0.014 | -0.015 | -0.014 | -0.013 | -0.012 | -0.013 | -0.013 | -0.011 | 0.000 |

B

|  | A1U | A2U | BOE | D2U | M16 | MN | POS | S1U | S3U | SBE | UA | X1 | X128 | X141 | X143 | X168 | X224 | X235 | X237 | X254 | X258 | X267 | X29 | X301 | X304 | X350 | X63 |
| --- | --- | --- | --- | --- | --- | --- | --- | --- | --- | --- | --- | --- | --- | --- | --- | --- | --- | --- | --- | --- | --- | --- | --- | --- | --- | --- | --- |
| A2U | 0.017 |  |  |  |  |  |  |  |  |  |  |  |  |  |  |  |  |  |  |  |  |  |  |  |  |  |  |
| BOE | 0.018 | 0.025 |  |  |  |  |  |  |  |  |  |  |  |  |  |  |  |  |  |  |  |  |  |  |  |  |  |
| D2U | 0.017 | **0.019** | 0.024 |  |  |  |  |  |  |  |  |  |  |  |  |  |  |  |  |  |  |  |  |  |  |  |  |
| M16 | 0.021 | **0.023** | **0.027** | 0.02 |  |  |  |  |  |  |  |  |  |  |  |  |  |  |  |  |  |  |  |  |  |  |  |
| MN | 0.065 | **0.061** | **0.068** | **0.065** | 0.06 |  |  |  |  |  |  |  |  |  |  |  |  |  |  |  |  |  |  |  |  |  |  |
| POS | **0.02** | **0.022** | **0.023** | 0.018 | **0.019** | 0.061 |  |  |  |  |  |  |  |  |  |  |  |  |  |  |  |  |  |  |  |  |  |
| S1U | **0.018** | 0.021 | **0.024** | 0.02 | **0.023** | **0.061** | 0.021 |  |  |  |  |  |  |  |  |  |  |  |  |  |  |  |  |  |  |  |  |
| S3U | 0.026 | **0.031** | **0.021** | 0.028 | **0.03** | **0.058** | 0.018 | 0.02 |  |  |  |  |  |  |  |  |  |  |  |  |  |  |  |  |  |  |  |
| SBE | 0.025 | **0.026** | **0.026** | 0.023 | 0.03 | **0.064** | **0.022** | **0.023** | 0.021 |  |  |  |  |  |  |  |  |  |  |  |  |  |  |  |  |  |  |
| UA | 0.03 | 0.027 | 0.032 | **0.027** | 0.031 | **0.054** | **0.031** | **0.03** | **0.033** | 0.038 |  |  |  |  |  |  |  |  |  |  |  |  |  |  |  |  |  |
| X1 | 0.016 | **0.022** | **0.02** | **0.019** | 0.022 | **0.059** | **0.017** | **0.019** | **0.016** | **0.013** | 0.033 |  |  |  |  |  |  |  |  |  |  |  |  |  |  |  |  |
| X128 | 0.022 | **0.023** | 0.024 | **0.02** | **0.021** | **0.057** | **0.019** | **0.014** | **0.022** | 0.023 | **0.029** | 0.018 |  |  |  |  |  |  |  |  |  |  |  |  |  |  |  |
| X141 | 0.015 | **0.016** | **0.018** | 0.015 | **0.021** | **0.06** | **0.018** | **0.019** | **0.025** | **0.021** | **0.028** | **0.016** | 0.017 |  |  |  |  |  |  |  |  |  |  |  |  |  |  |
| X143 | 0.016 | **0.02** | 0.024 | **0.016** | **0.021** | **0.062** | **0.02** | **0.017** | **0.023** | **0.028** | **0.026** | 0.021 | **0.02** | 0.017 |  |  |  |  |  |  |  |  |  |  |  |  |  |
| X168 | **0.024** | **0.023** | **0.028** | **0.022** | **0.023** | **0.054** | **0.022** | **0.021** | **0.027** | **0.033** | **0.021** | **0.026** | **0.021** | **0.023** | 0.018 |  |  |  |  |  |  |  |  |  |  |  |  |
| X224 | **0.023** | **0.021** | **0.028** | **0.019** | **0.021** | **0.05** | **0.019** | **0.022** | **0.025** | **0.027** | **0.015** | **0.024** | **0.021** | **0.022** | **0.018** | 0.013 |  |  |  |  |  |  |  |  |  |  |  |
| X235 | **0.027** | **0.026** | **0.03** | **0.025** | **0.025** | **0.052** | **0.026** | **0.025** | **0.028** | **0.031** | **0.022** | **0.028** | **0.024** | **0.026** | 0.026 | **0.023** | 0.015 |  |  |  |  |  |  |  |  |  |  |
| X237 | 0.024 | **0.021** | **0.025** | **0.02** | **0.019** | **0.05** | **0.021** | **0.02** | 0.024 | **0.03** | **0.022** | 0.024 | 0.019 | **0.021** | 0.018 | **0.016** | 0.016 | 0.018 |  |  |  |  |  |  |  |  |  |
| X254 | **0.019** | **0.017** | **0.024** | **0.02** | **0.017** | **0.046** | **0.019** | **0.017** | **0.022** | **0.026** | **0.019** | **0.02** | **0.018** | **0.018** | 0.017 | **0.015** | **0.011** | **0.018** | 0.014 |  |  |  |  |  |  |  |  |
| X258 | 0.029 | **0.027** | **0.033** | **0.026** | **0.028** | **0.058** | **0.027** | **0.029** | **0.036** | **0.036** | **0.012** | **0.032** | **0.029** | **0.029** | **0.027** | **0.019** | 0.014 | **0.021** | **0.024** | 0.019 |  |  |  |  |  |  |  |
| X267 | 0.034 | **0.032** | **0.038** | 0.028 | **0.032** | **0.058** | **0.033** | **0.03** | **0.035** | **0.039** | **0.014** | 0.034 | **0.032** | 0.032 | 0.027 | **0.019** | 0.015 | 0.023 | 0.024 | **0.022** | 0.014 |  |  |  |  |  |  |
| X29 | **0.024** | **0.023** | **0.028** | **0.024** | **0.021** | **0.056** | **0.026** | **0.025** | **0.028** | **0.035** | **0.021** | **0.026** | **0.025** | **0.023** | **0.021** | **0.016** | **0.016** | **0.02** | **0.018** | **0.017** | **0.021** | 0.023 |  |  |  |  |  |
| X301 | **0.018** | **0.017** | **0.024** | **0.021** | **0.02** | **0.048** | **0.02** | **0.02** | **0.024** | **0.024** | **0.016** | **0.021** | **0.019** | **0.019** | **0.016** | **0.015** | **0.01** | **0.019** | **0.015** | **0.013** | **0.015** | **0.017** | 0.014 |  |  |  |  |
| X304 | **0.017** | **0.018** | **0.024** | **0.022** | **0.019** | **0.048** | **0.02** | **0.021** | **0.024** | **0.028** | **0.026** | **0.023** | **0.019** | **0.021** | 0.021 | 0.02 | 0.018 | 0.023 | 0.016 | **0.017** | 0.024 | **0.027** | 0.021 | 0.015 |  |  |  |
| X350 | **0.017** | **0.019** | **0.021** | **0.017** | **0.024** | **0.063** | **0.019** | **0.012** | **0.021** | **0.023** | **0.027** | **0.016** | **0.018** | **0.016** | **0.021** | **0.024** | **0.022** | **0.026** | **0.022** | **0.019** | **0.027** | **0.032** | 0.024 | 0.02 | 0.021 |  |  |
| X63 | **0.017** | **0.016** | **0.022** | **0.019** | **0.018** | **0.05** | **0.019** | **0.021** | **0.025** | **0.025** | **0.019** | **0.021** | **0.019** | **0.017** | 0.015 | 0.016 | **0.014** | **0.02** | **0.013** | **0.014** | **0.018** | **0.021** | 0.014 | 0.012 | 0.017 | 0.022 |  |

|  |  |  |  |  |  |  |  |  |  |  |  |  |
| --- | --- | --- | --- | --- | --- | --- | --- | --- | --- | --- | --- | --- |
|  |  |  |  |  |  |  |  |  |  |  |  |  |
|  |  |  |  |  |  |  |  |  |  |  |  |  |
|  |  |  |  |  |  |  |  |  |  |  |  |  |
|  |  |  |  |  |  |  |  |  |  |  |  |  |
|  |  |  |  |  |  |  |  |  |  |  |  |  |
|  |  |  |  |  |  |  |  |  |  |  |  |  |
|  |  |  |  |  |  |  |  |  |  |  |  |  |
|  |  |  |  |  |  |  |  |  |  |  |  |  |
|  |  |  |  |  |  |  |  |  |  |  |  |  |
|  |  |  |  |  |  |  |  |  |  |  |  |  |
|  |  |  |  |  |  |  |  |  |  |  |  |  |
|  |  |  |  |  |  |  |  |  |  |  |  |  |
