# Supplementary material for: Micro- and Macro-Geographic Scale Effect on the Molecular Imprint of Selection and Adaptation in Norway Spruce
Source: PLoS One. 2014 Dec 31;9(12):e115499. doi: 10.1371/journal.pone.0115499 (PMC4281139; doi:10.1371/journal.pone.0115499)
Supplement: S3 Table — Analysis of variance at micro- and macro-geographic scales. F statistics were calculated at different levels using HIERFSTAT library in R: between transects or among clusters, among populations, among samples (A). AMOVA analysis calculated using Arlequin at micro- and macro-geographic scales. Fixation indexes statistically significant (*** P<0.000) (B). (DOC) [file pone.0115499.s006.doc]

**Table S3**: A) Analysis of variance at micro- and macro-geographic scales. *F* statistics were calculated at different levels using HIERFSTAT library in R: between transects or among clusters, among populations, among samples. B) AMOVA analysis calculated using Arlequin at micro- and macro-geographic scales. Fixation indexes statistically significant (*** *P* <0.000).

| **A** | | | | |
| --- | --- | --- | --- | --- |
| **Scale** |  | ***F* statistic** |  |  |
|  |  |  |  |  |
|  |  |  |  |  |
|  |  |  |  |  |
|  |  |  |  |  |
|  |  |  |  |  |
| Micro |  | between transect | among populations | among samples |
|  | total | 0.00016 | 0.00204 | 0.00032 |
|  | between transects | 0.00000 | 0.00188 | -0.00048 |
|  | among populations | 0.00000 | 0.00000 | -0.00236 |
|  |  |  |  |  |
| Macro |  | among clusters | among populations | among samples |
|  | total | 0.01624 | 0.03439 | 0.03813 |
|  | among clusters | 0.00000 | 0.01845 | 0.02226 |
|  | among populations | 0.00000 | 0.00000 | 0.00387 |

| **B** |  |  |  |  |  |  |  |
| --- | --- | --- | --- | --- | --- | --- | --- |
|  | **Source of variation** | **d.f.** | **Sum of squares** | **Variance compon.** | **Perc. of var.** | **Fixation index** | **Estimate** |
|  |  |  |  |  |  |  |  |
|  |  |  |  |  |  |  |  |
|  |  |  |  |  |  |  |  |
|  |  |  |  |  |  |  |  |
|  |  |  |  |  |  |  |  |
|  |  |  |  |  |  |  |  |
| Micro |  |  |  |  |  |  |  |
|  | between transects | 1 | 10.912 | -0.001 | 0 | *F*_CT_ | -0.0001 |
|  | among populations within transect | 10 | 112.903 | -0.328 | -1.2 | *F*_SC_ | -0.0119 |
|  | within populations | 590 | 16362.99 | 27.734 | 101.2 | *F*_ST_ | -0.0120 |
|  | Total | 601 | 16486.81 | 27.405 |  |  |  |
|  |  |  |  |  |  |  |  |
| Macro |  |  |  |  |  |  |  |
|  | among clusters | 3 | 425.498 | 0.4257 | 1.54 | *F*_CT_ | 0.0154*** |
|  | among populations within cluster | 23 | 922.767 | 0.3233 | 1.17 | *F*_SC_ | 0.0119*** |
|  | within populations | 1073 | 28826.649 | 26.865 | 97.29 | *F*_ST_ | 0.0271*** |
|  | Total | 1099 | 30174.915 | 27.615 |  |  |  |
